# An appeal to humanity: legal victory in favour of North America's only supervised injection facility: Insite

**DOI:** 10.1186/1477-7517-7-23

**Published:** 2010-10-09

**Authors:** Dan Small

**Affiliations:** 1 Director PHS Community Services Society Vancouver, Canada; 2 Department of Anthropology University of British Columbia Vancouver, Canada

## Abstract

Canada's federal government has once again failed to shut North America's only authorized supervised injection facility: Insite. A majority ruling issued by the BC Court of Appeal on 15 January 2010 upheld an earlier British Columbia Supreme Court ruling in 2008 that protected the rights of injection drug users (IDUs) to access Insite as a health facility as per the Charter of Rights and Freedoms component of the Constitution of Canada. The majority decision from Honourable Madam Justices Rowles, Huddart and Smith also established a jurisdictional victory safeguarding Insite as most appropriately run under the authority of the province of British Columbia rather than the federal Government of Canada. The Federal Government has appealed the case to the Supreme Court of Canada. A hearing date has been set for 12 May 2011. The appeal will be a legal one but even more so, it will be an appeal to humanity.

## 

Canada's federal government has once again failed to shut North America's only authorized supervised injection facility: Insite. A majority ruling issued by the BC Court of Appeal on 15 January 2010 upheld an earlier British Columbia Supreme Court ruling in 2008 that protected the rights of injection drug users (IDUs) to access Insite as a health facility as per the Charter of Rights and Freedoms component of the Constitution of Canada.

The majority decision from Honourable Madam Justices Rowles, Huddart and Smith also established an important jurisdictional victory emerging from the cross appeal by the operators of Insite: the PHS Community Services Society (PHS). The ruling further safeguards Insite as most appropriately run under the authority of province of British Columbia rather than the federal Government of Canada.

Insite opened on 21 September of 2003 under an exemption granting it status as a scientific pilot study until 12 September 2006. The primary goals of the program are: (1) to reach a marginalized group of IDUs with healthcare and supports who would otherwise be forced to use drugs in less safe settings (2) to reduce dangerous injection practices (syringe sharing) thereby reducing the risk of infectious diseases like HIV and HCV; and (3) to reduce fatal overdoses in the population of people that use the facility. The program also aims to provide referrals to treatment and detoxification, reduce public disorder (public injection) and validate the personhood of a deeply stigmatized target population[1].

The legal battle began near the end of Insite's three-year exemption for scientific study when a minority conservative government was elected in Canada on 6 February 2006. The new government voiced opposition to the program during and after the election[2-4]. On 1 September 2006, the program was given a temporary extension to operate until 31 December of 2007. Before this reprieve, the community waited in apprehension. The photograph below (Figure 1) shows an announcement of support for the supervised injection facility from a humble church in the inner city of Vancouver where Insite makes its home. This same church opens its pews up each night for the homeless to sleep and has held many services for local residents who died of preventable overdoses before Insite was opened. [insert figure here] For the people living with addictions and their families who face the dangers of preventable overdoses and infections in their everyday lives, the fate of the injection facility is neither academic nor legal. It is risk that is lived[5].

**Figure 1:**
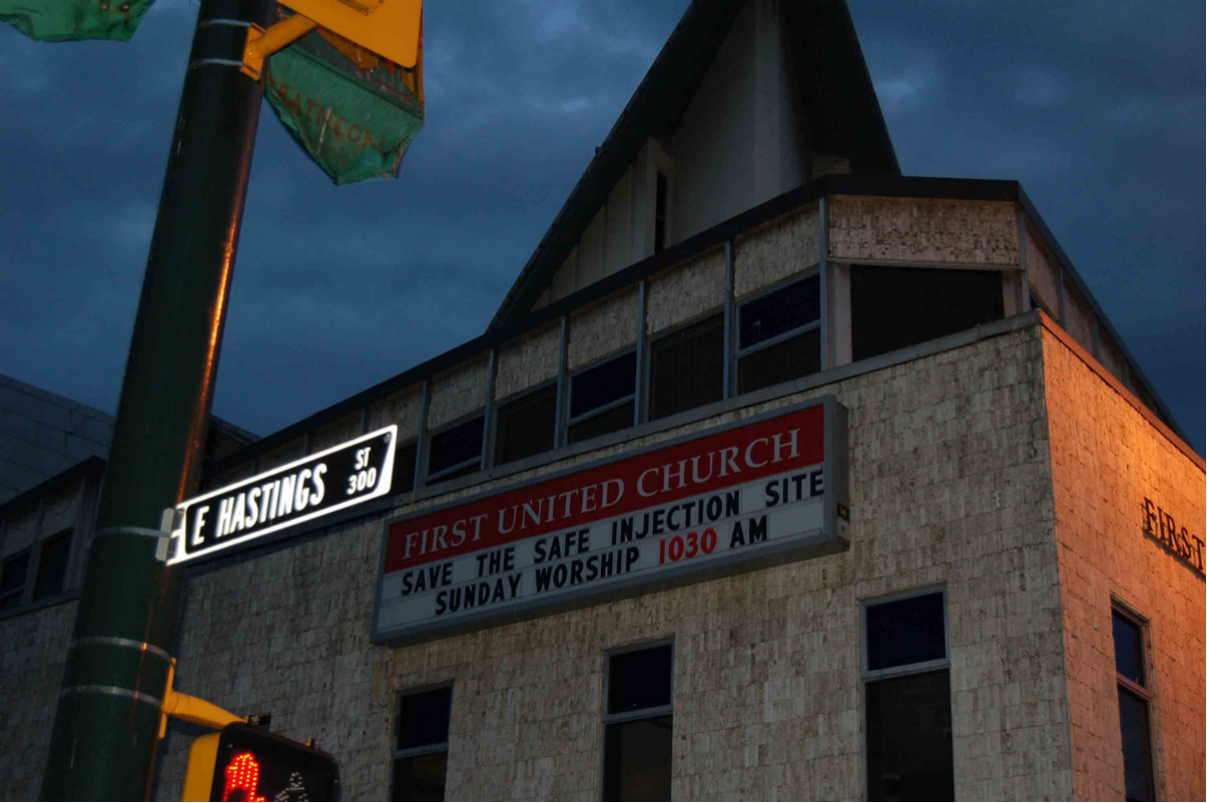
**Photograph of church marquee advertising an upcoming sermon in Vancouver's downtown eastside**. Photograph by D. Small

On 2 October 2007, the project was given an additional exemption to operate under the *Controlled Drugs and Substances Act *until 30 June 30 2008. A looming threat of closure by the conservative led government led the PHS to take the Government of Canada to court in late 2007[6]. The outcome of this first legal case determined that the *Controlled Drugs and Substances Act *(CDSA) in Canada is unconstitutional as it pertains to Insite because the closure of the program under the Act would impede people with addictions from receiving life saving healthcare. BC Supreme Court Justice Ian Pitfield ruled that the use of the CDSA to shut Insite would undermine the fundamental right, under Canada's Charter of Rights and Freedoms to life, liberty and security of the person[7].

Since its inception, Insite has been subject to an independent review by a team of physicians and scientists put in place to provide an "arm's length" evaluation of the program. The results of this scientific evaluation have been published in peer-reviewed academic journals and have indicated that Insite has reduced unsafe injection practices, public disorder, overdose deaths and HIV/Hepatitis while increasing uptake of addiction services and detox[8]. To date, there have been over three-dozen peer-reviewed papers evaluating Insite published making it one of the most evaluated healthcare programs in the history of Canada[9-38]. In light of the evidence, the program has garnered widespread support from Canadian physicians, scientists and healthcare professionals[39].

Despite this support from the scientific and medical community, the Conservative government of Canada remains entrenched in its position having served the PHS with court documents indicating their intention to appeal the case of Insite to the highest court in the country: the Supreme Court of Canada[40]. A court date to hear the case has been set for 12 May 2011 by the Supreme Court of Canada. It appears that science and ideology are once again at odds while Canada's highest court is asked to determine whether the earth is flat or round in the universe of addiction. When Insite reaches the end of its legal journey in Canada, hopefully the courts will once again rule that addiction is principally an issue for the Chief of Medicine rather than the Chief of Police. As a result, the case is more than an appeal to the Canada's highest court; it is an appeal to the country's humanity.

## Competing interests

The author declares that they have no competing interests.
